# Telephones in Hospitals: Rival Systems and Improvements Compared

**Published:** 1913-09-20

**Authors:** Humeral Condyle


### TELEPHONES IN HOSPITALS.

### Rival Systems and Improvements Compared.

By "HUMERAL CONDYLE."

A TELEPHONE system in any large institution is an admitted necessity. Yet, in some even of the best voluntary hospitals of the Metropolis, telephone economy is pushed almost to non-efficiency. Thus, up to quite recently, one telephone had to do duty at the busy Brompton Hospital for the use of the matron, the steward, and the resident medical officer. Now, however, an enterprising official has managed to get three telephones, one for each of these important officers.

A telephone system in any medical institution has to be considered from two points of view. First, the local telephone, with its miniature exchange, connecting different parts of the building; and, secondly, the system by which the building is put into communication with the world outside. These two installations may be considered separately.

A medical institution should be in direct communication with the public *via* the large district exchange. This is for the convenience of the institution as well as the public. The medical superintendent, the matron, the steward, the dispenser, all may have business necessitating telephonic communication. Urgent matters requiring immediate attention arise in all institutions. These facts are so obvious as scarcely to need comment. All the London voluntary hospitals and most of the infirmaries fulfil these requirements. There are, however, some which do not. The fever hospitals of the Metropolitan Asylums Board are poorly equipped telephonically. It seems incredible to the man in the street that he cannot get into direct communication with any of the Board's fever hospitals for any purpose whatever. All their telephones are linked up to an exchange at the Board's central offices at the Embankment, through which all communications must pass. The tyrannical centralisation policy of this Board is here exposed in its extreme form. Frequently one hospital is linked on to another, and sometimes even to a third, before a connection to the central offices is obtained. This involves a great waste of time. To illustrate this system we will take an example at random. If a communication has to be made to the Park Hospital (now used for children) one has to ring up the district exchange, then on to the Metropolitan Asylums Board Central Exchange, then on to the South-Eastern Ambulance Station, then on to the South-Eastern Hospital, and thence to the Park Hospital—a system of five relays—a cumbersome and absurd system, with the inevitable delay at each relay station.

Such a policy obtains on the ground of economy and (let it be whispered) for the purpose of discouraging inquiry at fever hospitals. Would it not be just as economical in the end and more efficient for each hospital to have an outside telephone system of its own? Telephone clerks at the Embankment Office alone cost the ratepayers about £200 a year. Such an amount would provide the rental of twenty ordinary telephones. One or two institutions, owing to the inconvenience of the existing arrangement, have had to be linked up to the Post Office telephone system, at the unlimited service rate. Such was the case this year at the Tooting Bec Asylum. The number of calls annually totalled 4,0C0. A record was kept for a period of five weeks, and of 362 calls the line was found to be engaged on 184 occasions. Each time a relay station was engaged the call had to be postponed.

Many a medical superintendent at the Board's fever hospitals has reported in favour of an ordinary direct telephone. One has repeated the request annually without success. It is related that one of the managers suggested that were such an installation in use the medical officers and staff would be utilising the instrument constantly for their private messages and pleasure. Such an opinion is evidence of the ignorant and biassed views which operate against harmony and institutional efficiency. If this opinion of the resident medical officer is general there is little surprise to be expressed at the difficulty of obtaining and retaining the services of the right type of men; but the abuse of the telephone is practically never observed in any institution so equipped, and, in any case, regulations can be made guarding against such a contingency.

This matter of a centralised telephone system is of some importance. In the event of a fire destroying the switchboard at the central offices, the whole fever administration of London would be thrown out of gear, for all the fever hospitals would be without telephonic communication.

Another point stated against such an installation is that the public and the friends and relatives of the sick would be continually pestering the institutions with inquiries, and that inquiries should never be answered by telephone, owing to the liability to mistakes; but surely a system that is good enough for the voluntary hospitals and the infirmaries ought to be good enough for the fever hospitals.

It is perhaps time that the managers of the Metropolitan Asylums Board should have their attention drawn to what amounts at times to a positive scandal, and be respectfully asked, for the sake of the long-suffering public as well as for the convenience of institutional administration, that a direct telephone system be installed in every institution under their management.

Reverting to the local purely institutional telephones, one or two recent innovations may be mentioned. Most institutions have the telephone with its bell in the corridor. The telephone instrument should be in the ward and the bell in the corridor—the former so that the nurse should be able to deliver or receive messages without leaving the ward, the latter so that the patients, especially at night, may not be disturbed by the noise.

Automatic telephones are now in use, and are likely in the near future to become general. At a recent conversazione held at King's College, Strand, among the many interesting appliances exhibited in the engineering department was an automatic telephone eminently suitable for use in medical institutions. It was being exhibited by permission of the Postmaster-General, who loaned a special technician to explain with demonstrations the working of the system. A similar automatic exchange and system has been working at Epsom for some months, with (it is said) excellent results. There can be no doubt as to its adaptability for institutions. It is simple to work, easy to understand, connects and disconnects automatically, buzzes the engaged signal readily, and saves time. A small exchange for 100 connections will be all the ordinary institution would require. The only difficulty would be its repair or retouching when out of gear, but this difficulty can be overcome. The appliance is worthy of an extended trial in an institution.

